# Locally advanced gallbladder cancer treated with effective chemotherapy and subsequent curative resection: a case report

**DOI:** 10.1186/s13256-021-03248-9

**Published:** 2022-01-17

**Authors:** Masashi Inoue, Keishi Hakoda, Hiroyuki Sawada, Ryuichi Hotta, Ichiro Ohmori, Kazuaki Miyamoto, Kazuhiro Toyota, Seiji Sadamoto, Tadateru Takahashi

**Affiliations:** 1 Department of Surgery, National Hospital Organization Higashihiroshima Medical Center, 513 Jike, Saijo-cho, Higashihiroshima, Hiroshima 739-0041 Japan; 2 Department of Gastrointestinal and Transplant Surgery, Applied Life Sciences, Institute of Biomedical and Health Sciences, Hiroshima University, Higashihiroshima, Hiroshima Japan

**Keywords:** Gallbladder cancer, Chemotherapy, Resection, Conversion surgery, Case report

## Abstract

**Background:**

Surgical resection of gallbladder cancer with negative margins is the only potentially curative therapy. Most patients with gallbladder cancer are diagnosed in an advanced stage and, despite the availability of several chemotherapies, the prognosis remains dismal. We report a case of locally advanced gallbladder cancer that was successfully treated with effective cisplatin plus gemcitabine, followed by curative resection.

**Case presentation:**

A 55-year-old Japanese female was hospitalized with right hypochondrial pain. Enhanced computed tomography revealed a 49 × 47 mm mass at the neck of the gallbladder, with suspected invasion of the liver and right hepatic artery. Endoscopic retrograde cholangiopancreatography demonstrated displacement of the upper bile duct. Intraductal ultrasonography showed irregular wall thickening and disappearance of the wall structure in bile ducts from the B4 branch to distal B2 and B3. Percutaneous transhepatic biliary biopsy revealed a poorly differentiated carcinoma. The patient was diagnosed with unresectable gallbladder cancer (T4N0M0 stage IVA). Cisplatin plus gemcitabine chemotherapy was initiated. After six courses of chemotherapy, enhanced computed tomography showed that the mass in the neck of the gallbladder had shrunk to 30 mm, Endoscopic retrograde cholangiopancreatography showed improvement of the hilar duct stenosis. A biopsy of the bile duct mucosa showed no malignant cells in the branch of the left and right hepatic ducts, the left hepatic duct, or the intrapancreatic ducts. The patient underwent conversion surgery with right and segment 4a liver resection, extrahepatic duct resection, and cholangiojejunostomy. The histopathologic diagnosis showed that the tumor cells had shrunk to 2 × 1 mm, and that R0 resection of the T2aN0M0 stage IIA tumor was successful.

**Conclusion:**

Although conversion surgery for gallbladder cancer is rarely possible, curative resection may offer a better prognosis, and it is important to regularly pursue possibilities for surgical resection even during chemotherapy.

## Introduction

Gallbladder cancer (GBC) is a rare disease accounting for 4% of all gastrointestinal malignancies, but there is a higher incidence in countries such as India, Japan, Chile, and Mexico [1, 2]. Due to the lack of early symptoms, most of the patients with GBC are diagnosed at advanced stage, in which the 5-year survival in less than 10% [2, 3]. Surgical resection of GBC)with negative margins is the only potentially curative therapy [2]. Despite the application of aggressive surgical approach and radical resections, the prognosis of GBC remains dismal [4].

Chemotherapy is the standard treatment for unresectable gallbladder cancer. Combination chemotherapies with gemcitabine and cisplatin (GC) or gemcitabine and S-1 (GS) are recommended as first-line chemotherapy regimens for nonresectable biliary tract cancer. Combination chemotherapy with gemcitabine, cisplatin, and S-1 (GCS) is also a candidate regimen [5]. On the other hand, there are few reports on conversion surgery after chemotherapy.

Herein, we report a case of locally advanced GBC that was successfully treated with effective cisplatin plus gemcitabine followed by curative resection.

## Case presentation

**Fig. 1:**
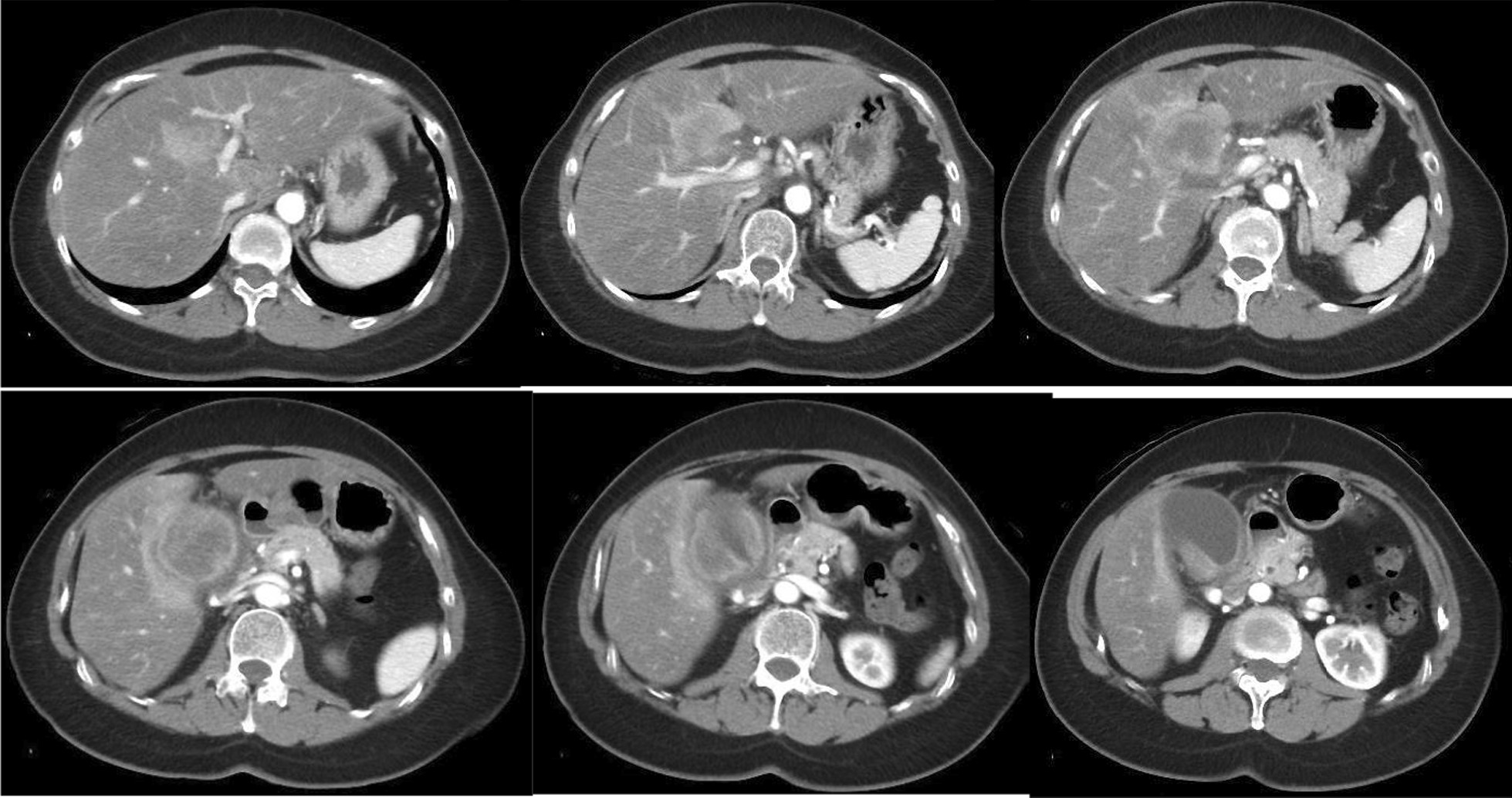
CT revealed a 49 × 47 mm mass at the neck of the gallbladder, with suspected infiltration of the liver and right hepatic artery.

**Fig. 2:**
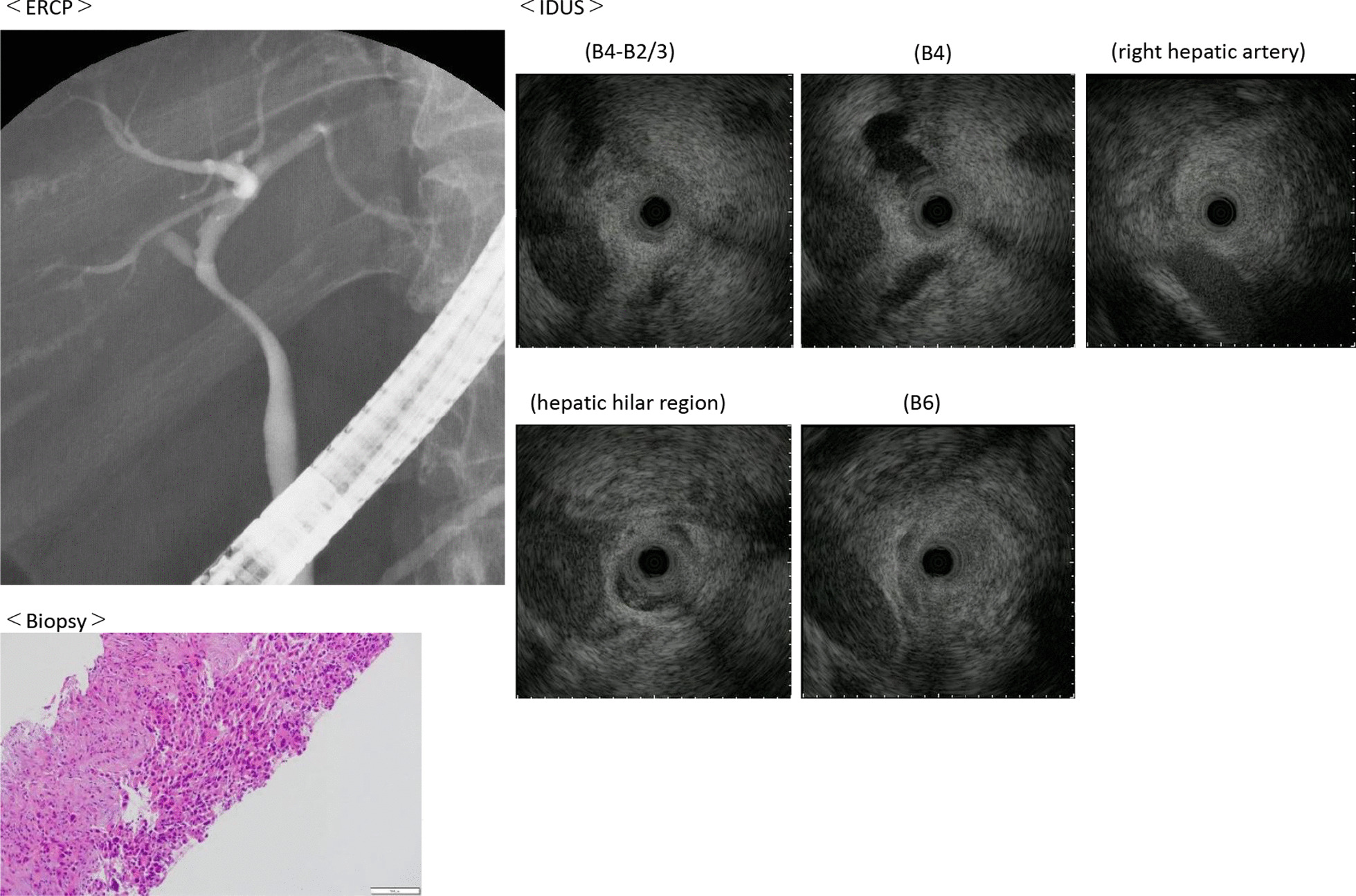
Endoscopic retrograde cholangiopancreatography revealed displacement of the upper bile duct. Intraductal ultrasonography showed irregular wall thickening and disappearance of the wall structure in bile ducts from the B4 branch to distal B2 and B3. Percutaneous transhepatic biliary biopsy revealed a poorly differentiated carcinoma.

**Fig. 3:**
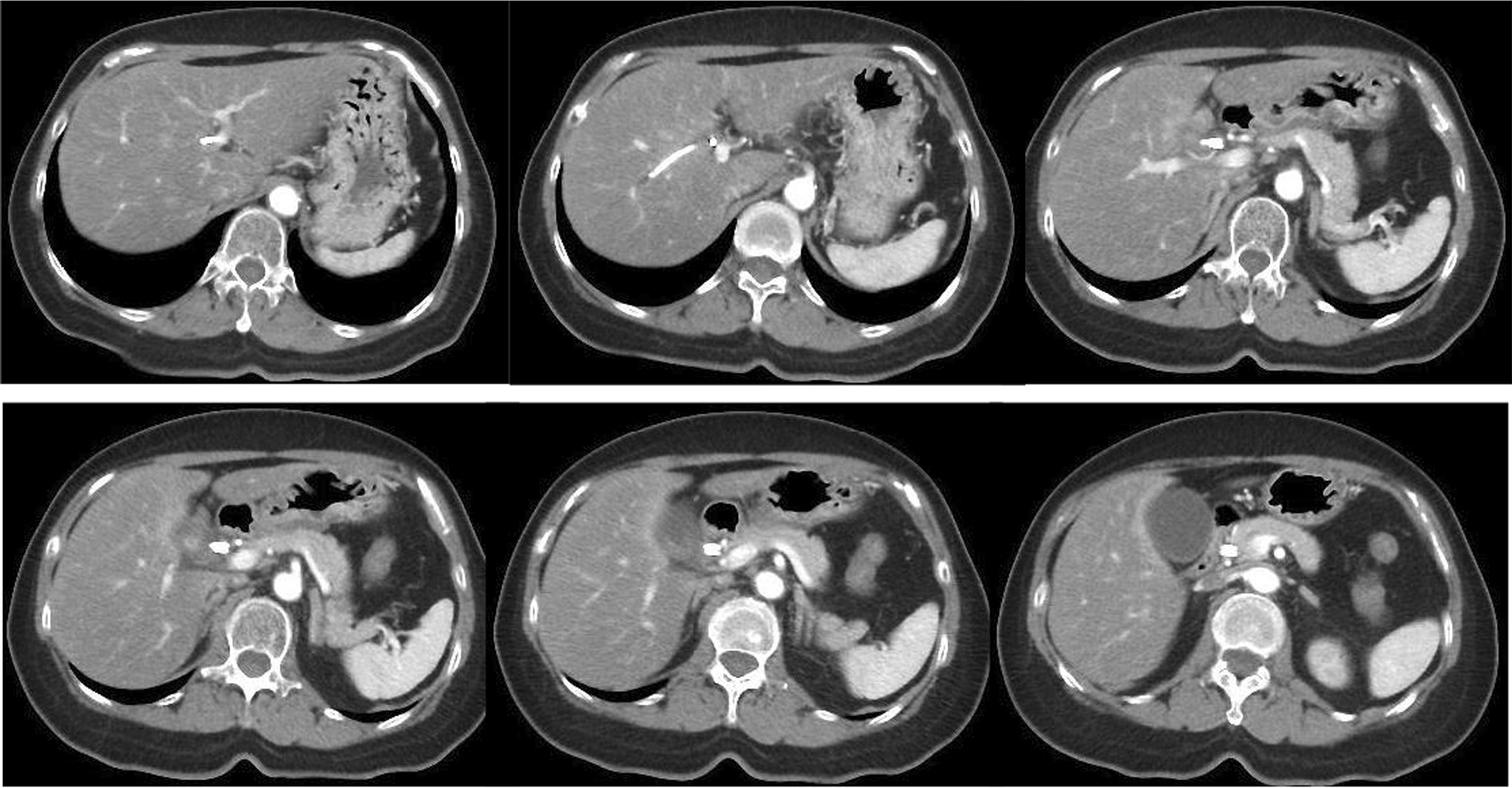
After six courses of chemotherapy, computed tomography examination showed that the mass in the neck of the gallbladder had shrunk to 3 cm, and endoscopic retrograde cholangiopancreatography showed improvement of hilar duct stenosis.

**Fig. 4:**
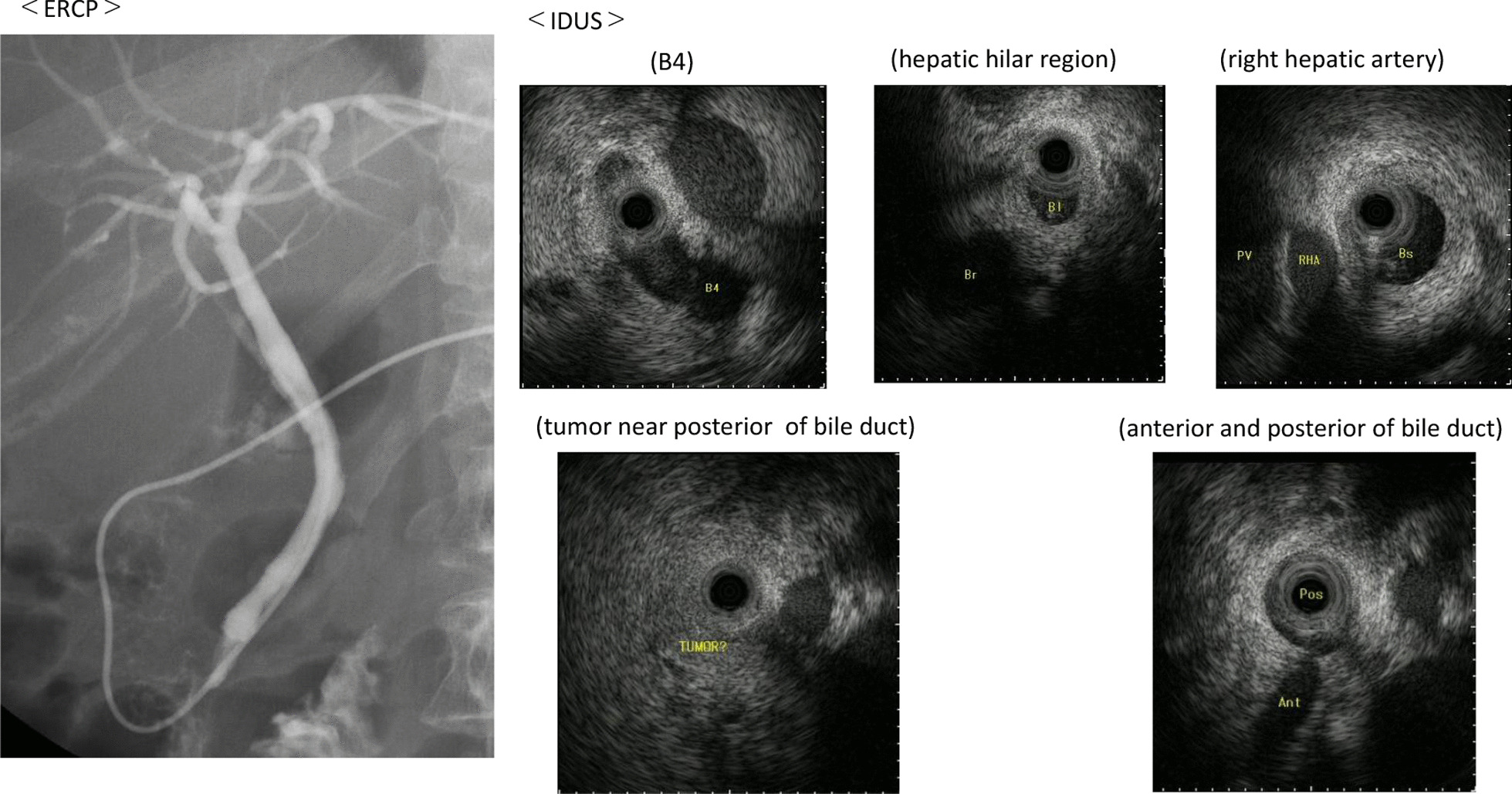
Intraductal ultrasonography revealed localized wall thickening in the extra-pancreatic ducts starting at the branch of the left and right hepatic ducts. Biopsy of bile duct mucosa found no malignant cells in the branch of the left and right hepatic ducts, the left hepatic duct, or the intrapancreatic ducts.

**Fig. 5:**
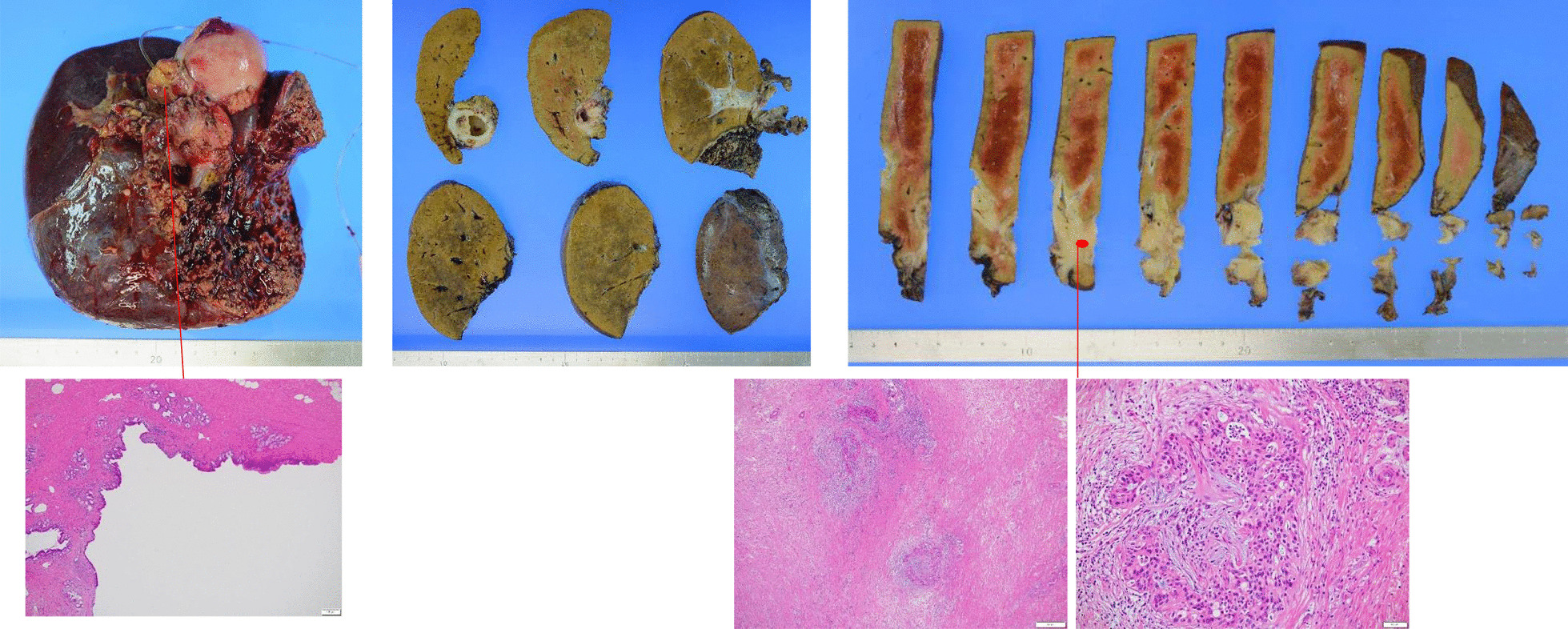
Histopathologic diagnosis found that the tumor cells had shrunk to 2 × 1 mm, and that R0 resection of the T2 (SS) N0M0 stage II tumor was successful.

A 55-year-old Japanese female was hospitalized with right hypochondrial pain. Laboratory tests showed elevated hepatobiliary enzymes and C-reactive protein. Serum levels of carcinoembryonic antigen and carbohydrate antigen 19-9 were 0.8 ng/ml and 3.2 U/ml, respectively. Enhanced computed tomography (eCT) imaging revealed a 49 × 47 mm mass at the neck of the gallbladder, with suspected invasion of the liver and right hepatic artery (Fig. 1). Endoscopic retrograde cholangiopancreatography (ERCP) demonstrated displacement of the upper bile duct. Intraductal ultrasonography (IDUS) showed irregular wall thickening and disappearance of the wall structure in bile ducts from the B4 branch to distal B2 and B3. Percutaneous transhepatic biliary biopsy revealed a poorly differentiated carcinoma (Fig. 2). The patient was diagnosed with unresectable gallbladder cancer [GBC (T4N0M0 stage IVA)]. Cisplatin plus gemcitabine chemotherapy was initiated. After six courses of chemotherapy without severe adverse events, a CT examination showed that the mass in the neck of the gallbladder had shrunk to 30 mm, ERCP showed improvement of the hilar duct stenosis (Fig. 3), and IDUS revealed localized wall thickening in the extra pancreatic ducts, starting at the branch of the left and right hepatic ducts. A biopsy of the bile duct mucosa showed no malignant cells in the branch of the left and right hepatic ducts, the left hepatic duct, or the intrapancreatic ducts (Fig. 4). The patient was scheduled for conversion surgery. The right hepatic artery was in close proximity to the tumor and involvement was suspected; the left hepatic artery was distant from the tumor. In addition, segment 4a was included in the resection range for gallbladder bed resection. Right and segment 4a liver resection and extrahepatic bile duct resection was scheduled. The residual liver capacity was 40% according to the planned surgical procedure. The indocyanine green (ICG) 15-minute value was 4.6% and the plasma clearance rate of ICG (ICGK) was 0.162. The future liver remnant ICGK (ICGK-F) was 0.065, which met the standard hepatectomy range at our hospital, and the ICGK-F was > 0.05; thus, surgery was performed. We performed a right liver/S4a resection, extrahepatic duct resection, and cholangiojejunostomy. The surgical findings showed no retention of ascites, peritoneal dissemination, or liver metastases. After Kocher maneuver, #16 lymph node was not swelling and did not dissect. The # 13a lymph node was dissected from the posterior surface of the pancreatic head. The anterior surface of the hepatoduodenal ligament was dissected, and the # 8 lymph node was dissected. The common bile duct was dissected at the upper edge of the duodenum and the pancreatic stump was sutured. The stump of the common bile duct was examined by immediate frozen-section analysis, and no malignant cells were found. The right hepatic artery, which ran on the dorsal side of the bile duct, was fixed by the bile duct and could not be dissected. The right hepatic artery was ligated and dissected at the bifurcation. The #12b and 12p lymph nodes were dissected, and the right branch of the portal vein was ligated and dissected at the bifurcation of the left and right portal veins. After right and S4a liver dissection was performed, the left bile duct and jejunum were anastomosed. The histopathologic diagnosis showed that the tumor cells had shrunk to 2 × 1 mm, and that R0 resection of the T2aN0M0 stage II tumor was successful. Residual tumor cells were found in a small part of the gallbladder neck and gallbladder wall at the hilum of the liver. The perimeter of the tumor cells was highly fibrotic, obscuring the layered structure of the gallbladder wall, gallbladder bed, and extrahepatic bile duct. We reasoned that the change was due to the disappearance of tumor cells associated with chemotherapy. Most of the gallbladder wall was highly fibrotic, with no residual tumor cells. No tumor cells were found in the mucosal epithelium of the cystic duct and intrahepatic extrahepatic bile duct. A high degree of fibrosis was observed near the right hepatic artery in the hilar region, but no infiltration of tumor cells was observed. No exposure of tumor cells was observed at the left intrahepatic bile duct stump (Fig. 5).

The patient was discharged from the hospital and there was no evidence of recurrence 12 months after surgery.

## Discussion

The curative treatment for biliary tract cancer is surgical resection, but biliary tract cancer can be unresectable due to a number of factors. As with other diseases, patient factors include a general health status that is a contraindication for surgical resection, and decreased hepatic reserve when hepatectomy is required. The standard value of future liver remnant function differs depending on the facility, and many facilities set the resectable limit by their own method [6, 7]. Among the tumor factors, cases with distant metastases are treated as unresectable [5]. Multiple intrahepatic metastases are often thought to be unresectable because the prognosis after resection is generally poor [8, 9]. The Bismuth–Corlette classification represents the affected site of the bile duct in hilar cholangiocarcinoma, but is often used to determine resectability [10, 11]. Bismuth IV, which extends to the secondary bifurcation (regional branch) of the left and right bile ducts, is considered unresectable [12, 13]. With respect to vascular invasion, bilateral portal vein invasion, hepatic artery invasion on the planned remnant liver side, and common hepatic artery invasion are also considered unresectable. [5] Some surgeons, however, believe that a three-segment resection of the liver and combined resection of the portal vein and hepatic artery can be performed [14–18]. Thus, there is not sufficient consensus on the limit of resection due to local extension [19–21].

In our case, eCT and IDUS suggested hepatic invasion, right hepatic artery invasion, and extension to the secondary bifurcation of the left bile duct, thus nonresectable GBC was diagnosed. Combination chemotherapies with gemcitabine and cisplatin (GC) or gemcitabine and S-1 (GS) are recommended as first-line chemotherapy regimens for nonresectable biliary tract cancer. Combination chemotherapy with gemcitabine, cisplatin, and S-1 (GCS) is also a candidate regimen [5]. In this case, GC therapy was selected. In the ABC-02 study, conducted in the United Kingdom, the median survival time of the gemcitabine monotherapy group and the gemcitabine and cisplatin combination therapy group was 8.1 and 11.7 months, respectively [22].

Since gemcitabine or S-1 have been administered to GBC, there have been case reports of conversion surgery. Based on a review of the PubMed database using the key words “gallbladder cancer,” “chemotherapy,” and “resection,” we found six cases of conversion therapy for initially unresectable locally advanced gallbladder cancer, including our case (Table 1) [23, 24]. Survival after surgery of three cases of R0 resection were from 6 to 42 months, and all cases were alive. On the other hand, duration of survival of three cases of R1 resection were from 8 to 19 months, and all cases were dead. Even in conversion surgery, achievement of curative resection was important for good prognosis.

**Table 1:** Reported cases of conversion therapy for initially unresectable locally advanced gallbladder cancer

Case	Year	Author	Age	Sex	Reasons for unresectability	Chemotherapy	Operation	Stage	Curability	Survival after surgery (months)	Status
1	2013	Kato	57	F	Arterial invasion	GEM	Right hepatectomy, caudate lobectomy, bile duct resection	IVa	R0	42	Alive
2	2013	Kato	57	F	Arterial invasion	GEM	Right hepatectomy, caudate lobectomy, bile duct resection	IVa	R1	18	Dead
3	2013	Kato	57	F	Arterial invasion, portal vein invasion	GEM	S4a + 5, bile duct resection	IVa	R1	19	Dead
4	2013	Kato	61	M	Arterial invasion	GEM	S4a + 5, bile duct resection	IVa	R1	8	Dead
5	2014	Einama	60	F	Arterial invasion	S-1	Right hepatectomy, bile duct resection	IVa	R0	30	Alive

Recently, neoadjuvant chemotherapy has been proposed, knowing the potential for an increase in the resectability rate and overall survival. Hakeem *et al*. [25] reported a systematic review involving 398 patients who were treated with neoadjuvant chemotherapy and 76 patients who received chemoradiotherapy. Of these patients, 50.4% were considered suitable for surgical resection and 191 (40.3%) underwent curative resection. The R0 rate for the entire cohort was 35.4%. The overall survival ranged from 18.5 to 50.1 months for patients who underwent curative resection versus 5.0–10.8 months for the nonresected group. However, this analysis is not enough to support the routine use of neoadjuvant therapy for GBC because the only patients with advanced GBC that may benefit from neoadjuvant therapy are those who will subsequently achieve an curative resection, accounting for only a third of the whole cohort.

In our case, eCT showed a marked reduction of the mass after six courses of GC therapy. An intrabile duct biopsy was performed by ERCP to rule out bile duct extension. The presence or absence of invasion to the right hepatic artery and hepatic invasion could not be evaluated. The liver remnant function was good and curative resection was considered possible by right and segment 4a liver resection and extrahepatic bile duct resection; thus, conversion surgery was performed. In the resected specimen, the tumor showed a marked shrinkage of approximately 2 mm. Residual tumor cells were found in a small part of the gallbladder neck and gallbladder wall at the hilum of the liver. No hepatic invasion, extrahepatic bile duct extension, or right hepatic artery invasion was observed. Limited surgery, such as liver segment 4a + 5 resection and extended cholecystectomy might have been possible while performing immediate frozen-section analysis, but the fibrosis that reflected the disappearance of tumor cells made it difficult to ablate the right hepatic artery. In GBC cases with suspected vascular invasion before chemotherapy, though chemotherapy is effective, surgery after chemotherapy may require major surgery.

This is a case of advanced GBC with a pathologically proven remarkable response to GC therapy.

This case is of a locally advanced GBC with marked tumor shrinkage with GC therapy. There are no reports that achieve a complete response from chemotherapy alone. Therefore, curative resection following GC therapy was important for the patient. There are few reports on conversion surgery for unresectable GB; therefore, the optimal chemotherapy regimen, preoperative period, and indications for curative surgery optimal surgical techniques are not clear. The short-term results, such as R0 resection rate and safety, and long-term results are also unclear. Further accumulation of cases is necessary.

## Conclusion

Although conversion surgery for GBC is rarely possible, curative resection may offer a better prognosis, and it is important to regularly pursue possibilities for surgical resection even during chemotherapy.

## Data Availability

Not applicable.

## Abbreviations

GBC: Gallbladder cancer
eCT: Enhanced computed tomography
ERCP: Endoscopic retrograde cholangiopancreatography
IDUS: Intraductal ultrasonography
ICG: Indocyanine green
ICGK: Plasma clearance rate of ICG
ICGK-F: Future liver remnant ICGK
GC: Gemcitabine and cisplatin
GS: Gemcitabine and S-1
GCS: Gemcitabine, cisplatin, and S-1
